# The learning curve for laparoscopic pancreaticoduodenectomy by a proficient laparoscopic surgeon: a retrospective study at a single center

**DOI:** 10.1186/s12893-023-02270-6

**Published:** 2024-01-03

**Authors:** Heng Wang, Xin Gao, Meng Liu, Xiaohan Kong, HongRui Sun, Zheyu Niu, Chaoqun Ma, Huaqiang Zhu, Jun Lu, Xu Zhou, Hengjun Gao, Faji Yang, Xie Song

**Affiliations:** 1 Department of Hepatobiliary Surgery, Shandong Provincial Hospital, Shandong University, Jinan, 250021 China; 2 Department of Hepatobiliary Surgery, Shandong Provincial Hospital, Shandong First Medical University, 324 Jingwuweiqi Road, Jinan, 250021 China; 3 Qilu Synva Pharmaceutical Co. Ltd, Dezhou, China; 4 Department of Physiology and Pathophysiology, School of Basic Medical Sciences, Cheeloo College of Medicine, Shandong University, Jinan, Shandong China

**Keywords:** Laparoscopic pancreatoduodenectomy, The cumulative sum method, Learning curve, Laparoscopic surgery

## Abstract

**Background:**

To explore the learning curve of single center laparoscopic pancreaticoduodenectomy (LPD) and evaluate the safety and efficacy of the operation at different stages.

**Methods:**

A detailed review was conducted on the clinical data of 120 cases of laparoscopic pancreatoduodenectomy performed by the same surgeon between June 2018 and June 2022. Cases that did not provide insights into the learning curve of the procedure were excluded. The cumulative sum (CUSUM) analysis and the best fitting curve methods were employed to delineate the learning curve based on operation time and intraoperative blood loss. The study further evaluated the number of surgeries required to traverse the learning curve. Outcome measures, including operation time, intraoperative blood loss, length of stay, complications, and other relevant indicators, were extracted and compared across different phases of the learning curve.

**Result:**

The maximum turning point of the fitting curve was found in 35 cases by the cumulative sum method of operation time, after which the learning curve could be considered to have passed. The fitting curve obtained by the cumulative sum method of intraoperative blood loss was stable in 30 cases and proficient in 60 cases, which was basically consistent with the fitting curve of operation time. Taking 35 cases as the boundary, the learning curve is divided into learning improvement stage and mastering stage. There was no statistical significance in the general data of the two stage patients (*P* > 0.05). Hospitalization days decreased from 19 to 15 days (*P* < 0.05);Pancreatic fistula decreased from 20.0% of grade B and 8.6% of grade C to 7.1% of grade B and 3.5% of grade C (*P* < 0.05), and the operative time decreased from (376.9 ± 48.2) minutes to (294.4 ± 18.7) minutes (*P* < 0.05). Intraoperative blood loss decreased from 375 to 241 ml (*P* < 0.05).

**Conclusion:**

Thirty-five patients with LPD can reach the proficiency stage and the perioperative indexes can be improved.

## Introduction

Laparoscopic pancreaticoduodenectomy (LPD) started in 1994 and is one of the most complex operations in general surgery [1]. With the development of endoscopic technology and the improvement of surgical level, more and more centers began to carry out LPD. Compared with open surgery, laparoscopic surgery has the advantages of less trauma, less intraoperative bleeding, shorter hospital stay, and faster postoperative recovery of patients [2, 3]. But because of the complex structure around the pancreas, limited endoscopic field of view, limited operating angle and many other problems [4], LPD is only performed in some large and experienced medical centers. Compared with open pancreaticoduodenectomy (OPD), the efficacy of LPD has gradually become a focus of attention and debate among surgeons [5, 6]. The mainstream view is that LPD has obvious advantages over OPD surgery [7], but there are also experts who offer the opposite view [8]. Multi-center studies have shown that in the early stage of LPD surgery, the short-term efficacy and prognosis of patients may not be ideal [9, 10], which may be due to the neglect of the influence of the learning curve in the LPD surgery process. Therefore, it is of great significance to analyze and understand the learning curve during LPD surgery to guide surgeons to carry out surgery smoothly and reduce surgical complications. In this paper, the clinical data of 120 patients with LPD in a single center were retrospectively analyzed to explore the learning curve and evaluate the safety and efficacy of surgery at different stages.

## Data and methods

### General information

The clinical data of patients undergoing laparoscopic pancreaticoduodenectomy in the Department of Hepatobiliary Surgery, Shandong Provincial Hospital from June 2018 to June 2022 were retrospectively analyzed. For all patients scheduled for a pancreaticoduodenectomy, an attempt was first made to perform the procedure using laparoscopic techniques. However, if during the operation it was found that tumor exposure was difficult, or there were challenges with adhesion separation, making the laparoscopic procedure difficult to carry out, then a conversion to an open pancreaticoduodenectomy would be made. Inclusion criteria: (1) Successful operation without laparotomy; (2) Preoperative imaging showed tumors around the pancreas head and ampulla without distant metastasis. Exclusion criteria: (1) absence of perioperative data; (2) conversion to laparotomy.

### Related to surgery

(1) Surgical methods: We adopted a comprehensive surgical approach for pancreaticoduodenectomy, using the 5 ports method to establish abdominal operating holes. Key procedures involved the exposure of the pancreas, dissection of various vessels and ligaments, and anastomosis of pancreatic duct, hepatic duct, and jejunum. Detailed steps of the surgical procedure can be found in reference [11]. (2) Intraoperative indicators: the time from the beginning of skin resection to the end of suture was calculated as the operating time, and the amount of intraoperative blood loss was recorded. (3) Postoperative indicators: incidence of pancreatic fistula, biliary fistula, gastric fistula, gastroparesis, postoperative exhaust and feeding time, postoperative bleeding, abdominal infection, reoperation and perioperative death were recorded. Perioperative time was defined as the time between admission and discharge. Postoperative pancreatic fistula (POPF) was defined according to the 2016 update of the International Study Group on Pancreatic Surgery (ISGPS) [12–14].

### CUSUM analysis was used to construct the learning fitting curve

CUSUM analysis, a time-weighted control graph method, was employed to plot the learning curve. It calculates deviations between observed and target values, accumulating as CUSUM: $\text{CUSUM}={\sum }_{\text{i=1}}^{\text{n}}\text{(Xi-u)}$,“Xi” represents the observed value for each patient (using operative time and intraoperative blood loss), “n” represents the surgical sequence number, “u” represents the mean value of this observation. The surgical sequence is plotted against CUSUM values, aiming for a fit coefficient ${R}^{2}$ closest to 1, indicating optimal model fit [15].

### Statistical processing

SPSS25.0 software was used for statistical analysis. The learning curve is drawn using R software version 4.1.2. Measurement data conforming to the normal distribution use $\left(\overline{x}\pm s\right)$ representation, and *t-* test was used for comparison between groups. Measurement data with non-normal distributions were represented by *M(Q1,Q3)*, and the rank sum test was used for comparison between groups. Frequency data (classified data) is represented by the number of cases (%), and comparison between groups using ${x}^{2}$ test or Fisher's exact probability method. All results were considered statistically significant with *P* < 0.05.

## Results

General information: Out of the 120 patients who underwent laparoscopic pancreatoduodenectomy, 69 were male and 51 were female, with an average age of (59.5 ± 8.9) years. A total of 25 patients were diagnosed with pancreatic cancer. It is noteworthy that no conversion to laparotomy was performed during the procedure. For further details, please refer to Table 1.

**Table 1:** Basic clinical features and baseline of patients with LPD

Clinical factor	LPD (*n* = 120)	
gender		
male	69 (57.5%)	
female	51 (42.5%)	
Age (years)	59.5 ± 8.9	
Main admission symptom		
jaundice	79 (65.8%)	
Abdominal pain	15 (12.5%)	
other	26 (21.7%)	
hypertension		
no	103 (85.8%)	
yes	17 (14.2%)	
diabetes		
no	112 (93.3%)	
yes	8 (6.7%)	
Smoking history		
no	81 (67.5%)	
yes	39 (32.5%)	
Drinking history		
no	89 (74.2%)	
yes	31 (25.8%)	
History of pancreatitis		
no	116 (96.7%)	
yes	4 (3.3%)	
History of abdominal operation		
no	105 (87.5%)	
yes	15 (12.5%)	
Major tumor size (cm)	2.5 ± 0.9	
American Society of Anesthesiologists (ASA) score		
≤ 2	73 (60.8%)	
> 2	47 (39.2%)	
Pathological findings		
cholangiocarcinoma	40 (33.3%)	
Periampullary carcinoma	16 (13.3%)	
Pancreatic cancer	25 (20.8%)	
Carcinoma of duodenal papilla	32 (26.7%)	
Pancreatic neuroendocrine carcinoma	7 (5.8%)	
Days from surgery to discharge (days)		16.2 ± 3.7
Operation time (minutes)		318.5 ± 48.2
Intraoperative blood loss (ml)		280.4 ± 159.2
Anal exhaust time (days)		2.8 ± 0.8
Postoperative feeding time (days)		2.9 ± 0.8

**Fig. 1:**
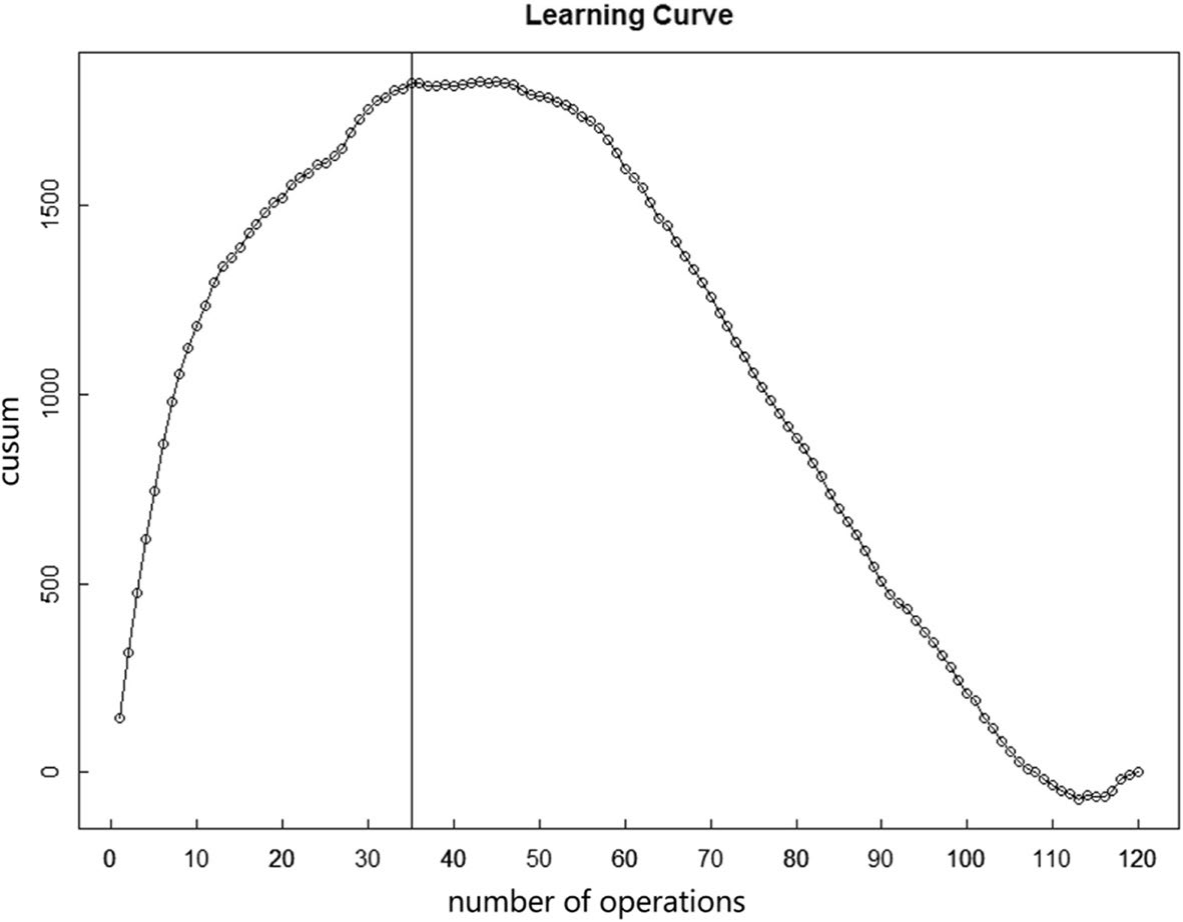
CUSUM learning curve of operative time

**Fig. 2:**
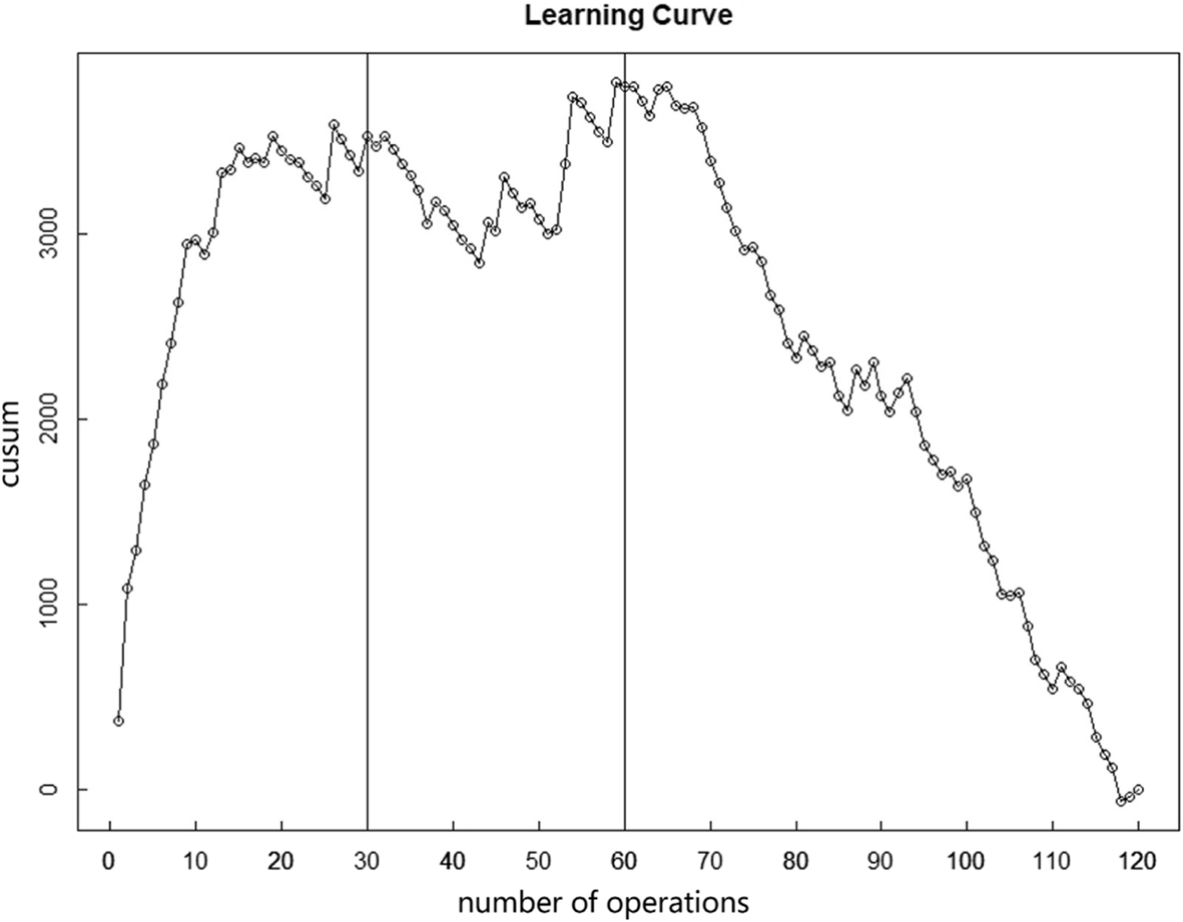
CUSUM learning curve of intraoperative blood loss

Results of the CUSUM learning curve analysis: Surgical times and intraoperative blood loss were statistically compiled for 120 patients. The cumulative sum (CUSUM) was determined using a summation approach, as detailed in Figs. 1 and 2. Following the formula: $\mathrm{CUSUM}={\sum }_{\text{i=1}}^{\text{n}}\text{(Xi-u)}$. A curve was plotted with the surgical sequence number on the x-axis and the CUSUM value on the y-axis. The surgical time curve reached its peak at the 35th case, while the curve for intraoperative blood loss stabilized around the 30th case and was considered proficiently mastered by the 60th case. These findings closely mirror the results derived from the surgical time data. Taking the 35th case as a milestone, cases 1–35 are categorized as the LPD learning and improvement phase, whereas cases from the 35th onward are viewed as the proficient application phase.

Comparison of general data in the two stages: General data in the two stages, including gender, age, admission symptoms, hypertension, diabetes, smoking history, drinking history and postoperative pathological types, were not statistically significant, as shown in Table 2.

**Table 2:** Comparison of general data of patients in the two stages

		Learning improvement stage (*n* = 35)	Proficiency stage (*n* = 85)	*P* value
gender				0.446
	male	22 (62.9%)	47 (55.3%)	
	female	13 (37.1%)	38 (44.7%)	
Age (years)		58.1 ± 8.8	60.0 ± 8.9	0.245
Main admission symptom				0.352
	jaundice	25 (71.4%)	54 (63.5%)	
	abdominal pain	2 (5.7%)	13 (15.3%)	
	others	8 (22.9%)	18 (21.7%)	
hypertension				0.581
	no	31 (88.6%)	72 (84.7%)	
	yes	4 (11.4%)	13 (15.3%)	
Diabetes mellitus				0.788
	no	33 (94.3%)	79 (92.9%)	
	yes	2 (5.7%)	6 (7.1%)	
smoking history				0.308
	no	26 (74.3%)	55 (64.7%)	
	yes	9 (25.7%)	30 (35.3%)	
Drinking history				0.633
	no	33 (94.1%)	62 (72.9%)	
	yes	8 (22.9%)	23 (27.1%)	
History of pancreatitis				0.351
	no	33 (94.3%)	83 (97.6%)	
	yes	2 (5.7%)	2 (2.4%)	
History of cholangitis				
	no	34 (97.1%)	84 (98.8%)	0.517
	yes	1 (2.9%)	1 (1.2%)	
history of abdominal operation				0.704
	no	30 (85.7%)	75 (88.2%)	
	yes	5 (14.3%)	10 (11.8%)	
Main tumor size (cm)		2.4 ± 1.1	2.5 ± 0.9	0.351
American Society of Anesthesiologists (ASA) score				(0.599)
	≤ 2	20 (57.1%)	53 (62.3%)	
	> 2	15 (42.9%)	32 (37.7%)	
pathological findings				0.377
	cholangiocarcinoma	11 (31.4%)	29 (34.1%)	
	Periampullary carcinoma	7 (20.0%)	9 (10.6%)	
	pancreatic cancer	4 (11.4%)	21 (24.7%)	
	Carcinoma of duodenal papilla	11 (31.4%)	21 (24.7%)	
	Pancreatic neuroendocrine cancer	2 (5.7%)	5 (5.9%)	
Body mass index()		18.5 ± 2.8	18.4 ± 2.7	0.909

Comparison of perioperative effects between the two stages:

In the LPD Learning Improvement Stage, the days from postoperative to discharge were 19, the operation time was 376.9 ± 48.2 min, and the intraoperative blood loss was 375 ml. The rates of Grade B and C pancreatic fistula were 20.0% and 8.6%, respectively.

In contrast, during the Proficient Application Stage, the days from postoperative to discharge reduced to 15, the operation time shortened to 294.4 ± 18.7 min, and the intraoperative blood loss decreased to 241 ml. The rates of Grade B and C pancreatic fistula reduced to 7.1% and 3.5%, respectively.

There were significant differences in days to discharge, operation time, intraoperative blood loss, and pancreatic fistula rates between the two stages (*P* < 0.05). However, biliary fistula, gastrointestinal fistula, gastroparesis, postoperative bleeding, reoperation, and perioperative death showed no significant differences between the groups (*P* > 0.05). Detailed data can be found in Table 3.

**Table 3:** Comparison of perioperative effects between the two stages

Clinical factor	Mode of operation		*P* value
	Learning improvement stage (*n* = 35)	*Proficiency stage (n = 75)*	
Days from surgery to discharge (days)	19.4 ± 2.6	*14.9 ± 3.2*	< 0.05
Operation time (minutes)	376.9 ± 48.2	294.4 ± 18.7	< 0.05
Intraoperative blood loss (ml)	375.1 ± 194.3	241 ± 123.8	< 0.05
Pancreatic fistula			0.049
No pancreatic fistula or biochemical leakage	25 (71.4%)	76 (89.4%)	
Grade B pancreatic fistula	7 (20.0%)	6 (7.1%)	
Grade C pancreatic fistula	3 (8.6%)	3 (3.5%)	
Biliary fistula			0.094
no	29 (82.9%)	79 (92.9%)	
yes	6 (17.1%)	6 (7.1%)	
Gastrointestinal fistula			0.972
no	33 (94.3%)	80 (94.1%)	
yes	2 (5.7%)	5 (5.9%)	
gastroplegia			0.351
no	33 (94.3%)	83 (97.6%)	
yes	2 (5.7%)	2 (2.4%)	
Anal exhaust time (days)	3.1 ± 0.9	2.7 ± 0.8	0.027
Postoperative feeding time (days)	3.3 ± 0.8	2.7 ± 0.8	0.001
Postoperative bleeding			0.070
no	30 (85.7%)	81 (95.3%)	
yes	5 (14.3%)	4 (4.7%)	
abdominal infection			0.008
no	28 (80.0%)	81 (95.3%)	
yes	7 (20.0%)	4 (4.7%)	
Reoperation			0.412
no	30 (85.7%)	80 (94.1%)	
yes	5 (14.3%)	5 (5.9%)	
Perioperative death			0.852
no	33 (97.1%)	85 (98.8%)	
yes	1 (2.9%)	1 (1.2%)	

## Discussion

After extensive development, endoscopic surgery has become a widely used treatment for tumors in various organs, with recognized safety and advantages. LPD was first published in 1994, but its development has been relatively slow. In recent years, LPD has been widely performed in major medical centers, but its safety remains controversial, and there is no consensus reached in clinical practice due to frequent anastomotic reconstructions, critical postoperative complications, concerns about a lack of radical curative effect in malignant tumors, and limitations in endoscopy technology [16]. While our study aligns with previous findings on the challenges and benefits of LPD, it offers unique insights into the learning curve associated with this procedure [17, 18]. Our data indicates a clear learning curve for surgeons undertaking LPD. This observation resonates with the findings of Jennifer F Tseng, who noted improvement in operation time and other parameters after surgeons accumulated experience with 60 PD cases [19]. However, it's important to highlight that our study observed this learning curve in the context of a single center, possibly leading to variations in outcomes compared to multi-center studies. Mohamed Abdelgadir Adam pointed out that the incidence of postoperative perioperative complications such as pancreatic fistula and postoperative bleeding in the LPD group was higher than that in the OPD group [20], possibly because the surgeons had not yet crossed the learning curve.

Studies both domestic and international reveal varying learning curves across different centers. Some centers have indicated a case number of 40 throughout the learning curve [21], while others point to multi-center studies with a case number of 49, where postoperative pancreatic fistula incidence appears to decrease at different stages of the learning curve [9]. Currently, an increasing number of medical centers are interested in performing laparoscopic pancreaticoduodenectomy (LPD), resulting in a rise in the number of procedures executed annually. However, the procedure remains challenging and carries potential risks. Acknowledging the presence of a learning curve is pivotal in guiding surgeons to progress from skill enhancement to mastery. While numerous studies have been conducted on LPD learning curves both domestically and internationally, few offer reference significance for individual centers or surgeons. Additionally, most studies involve surgeons with limited experience in laparoscopic surgery prior to LPD, potentially due to a shortage of high-volume research centers. There is a scarcity of data on the learning curve for LPD performed by surgeons with thousands of prior laparoscopic surgeries. This may explain why the surgeon in the current study required fewer procedures to surmount the learning curve and signifies that having adequate laparoscopic surgical experience can potentially shorten the learning curve before performing LPD. Variations in patient demographics, perioperative care protocols, and hospital levels may cause learning curves to differ among centers. A critical observation from our study was the influence of a surgeon's prior experience in laparoscopic surgeries on the learning curve of LPD. Our data suggests that surgeons with extensive laparoscopic experience might require fewer procedures to navigate the LPD learning curve efficiently. This finding underscores the value of comprehensive laparoscopic training for surgeons venturing into more complex procedures like LPD.

Numerous studies have reported on the learning curve of LPD. However, the customary broken-line chart is limited to operation time and the inflection point of the chart is often deemed as the cut-off point of the learning phase. Regrettably, this approach is highly subjective and lacks statistical support as it overlooks the influence of the patients' preoperative conditions on the operation duration [4, 22]. In this study, the CUSUM method was employed to establish a learning curve. After accurately fitting the curve, multiple regression analysis was conducted to eliminate the impact of patients' preoperative factors on operating time and obtain the benchmark operation duration. Ultimately, 35 patients were selected as nodes, which were verified by the learning curve of intraoperative blood loss. The CUSUM-derived learning curve not only highlights the inflection point of the operating time's descent but also considers whether the benchmark operating duration continues to decrease beyond the inflection point.

The findings of this study indicate that during the progression from the stage of improved learning to that of skilled application, the duration of operation gradually decreases while the incidence of pancreatic fistula shows a corresponding reduction. The statistical analysis of these results is significant, and reveals a correlation between operation time and short-term complications, in contrast to the conclusions drawn by Wang's investigation [16]. With the advancements in technical proficiency, the volume of intraoperative blood loss has gradually reduced. The adoption of the ERAS concept has resulted in a shortened postoperative feeding period. With the increased understanding of postoperative pancreatic fistula and biliary leakage and the refinement of treatment techniques, the percentage of abdominal infections during the skilled application phase has decreased. There were no significant differences in other perioperative complications such as postoperative bleeding and reoperation between the two groups. Currently, statistical data on the long-term survival rate and progression-free survival rate of patients after LPD is not available in our center due to the relatively short amount of time since we began performing the procedure. Further studies and continued follow-ups will enable us to determine the long-term efficacy of LPD.

However, our study is not without its limitations: the use of the CUSUM method for group categorization introduces an element of subjectivity, which might influence the outcomes. The criteria used to evaluate a surgeon's experience, particularly in the context of LPD, were not explicitly defined, leading to potential ambiguities in interpreting the learning curve. While our study offers valuable insights into the learning curve of LPD, it's essential to recognize that these findings might not be universally applicable, given the variations in patient demographics, perioperative care protocols, and hospital infrastructures across different centers. Furthermore, with the introduction of ERAS (Enhanced Recovery After Surgery), there might be an impact on the patient's length of hospital stay, potentially compromising the accuracy of our research concerning the duration of hospitalization.

The learning curve of a single center and surgeon discussed in this study may differ from the findings of other centers. Future multi-center and large sample learning curve analyses are expected to provide more valuable information for surgeons performing LPD surgeries.

## Conclusion

By utilizing CUSUM analysis, it has been concluded that experienced laparoscopic surgeons tend to reach the maximum turning point of their learning curve for operation time around the 35th case. Moreover, the curve fitting of intraoperative blood loss is relatively stable at 30 cases and proficient at 60 cases, which is lower than the typical number of operations needed to surpass the learning curve in the global medical community.

## Data Availability

The datasets and analytical data utilized in this experiment are not currently publicly available, but they may be made accessible to corresponding authors upon request.

## Abbreviations

LPD: Laparoscopic pancreaticoduodenectomy
CUSUM: Cumulative sum
OPD: Open pancreaticoduodenectomy
BL: Biochemical leakage
